# Caustic stenosis of the esophagus and malignant neoplasia: A dilemma

**DOI:** 10.3389/fonc.2022.1059524

**Published:** 2022-11-11

**Authors:** Nelson Adami Andreollo, Valdir Tercioti Jr, João de Souza Coelho Neto, José Antonio Possatto Ferrer, Luiz Roberto Lopes

**Affiliations:** Department of Surgery and Gastrocentro, Faculty of Medical Sciences, State University of Campinas – Unicamp, Campinas, Sao Paulo, Brazil

**Keywords:** caustics, dysphagia, esophageal stenosis, endoscopy, esophageal neoplasms

## Introduction

Caustic ingestion remains a complex public health issue worldwide, in adults and children. Caustic agent (acids and alkalis) is a product that causes tissue damage, and its ingestion will damage the mouth, pharynx, hypopharynx, esophagus, stomach, duodenum, and may reach the jejunum. Concomitant gastric and intestinal injuries can occur in 20 to 60% (1–3)

Acid products, most of the times, cause coagulative necrosis of the mucosa, while alkalis cause a liquefaction/saponification effect on the mucosa, reaching the entire wall of the organ. The most accessible acids are hydrochloric, sulfuric and oxalic acid, widely used in civil construction and factories. Products that contain alkali are those used in household cleaning, containing sodium hydroxide, also known as caustic soda. Therefore, caustic stenosis of the esophagus depends on the product ingested, the amount that was ingested and its concentration (1, 4–6).

Ingestion of these products damages the esophagus, in different extensions, and the final consequence is progression to stenosis and fibrosis of the organ. The most common symptom is dysphagia, and the intensity of dysphagia depends on the degree of stenosis. Odynophagia is also present. The nutritional status injury is evident, due to significant weight loss (1, 2, 4, 7).

Developing countries have a higher incidence of this kind of accident. In children under 10 years of age, ingestion is most often accidental, however, in adolescents and adults, the etiology of ingestion is due to attempted suicide. In the United States, despite the decline in caustic intake in children (5,000 to 15,000 per year), the incidence is 15.8 cases per 100,000 peoples (8). And the American Association of Poison Control (AAPCC), recorded that approximately 200.000 peoples were exposed to cleaning substances, as items of household use, including caustic products, since 2000 (3).

The treatment of esophageal strictures secondary to the ingestion of caustic products will depend on the extent of esophageal and gastrointestinal involvement, the degree of dysphagia caused to the patient and the clinical, systemic, nutritional repercussions and comorbidities. It is also important to assess gastric involvement, because gastric mucosal injury can lead to antropyloric stenosis. Zargar et al. observed that acute gastric injury was present in 85.4% of their patients who ingested acid, mainly involving the distal half of the stomach, with 44.4% presenting late complications, like pyloric or antral stenosis (7, 8).

The first treatment attempt to relieve dysphagia is esophageal dilatation with the aid of digestive endoscopy, which can be repeatedly performed (2, 4, 9). If there is no improvement in dysphagia, weight gain and the patient’s ability to ingest food orally, the surgical treatment is indicated. The recommended surgical procedure is retrosternal esophagocoloplasty, with better long-term results. Esophagogastroplasty, with an isoperistaltic gastric tube, is also indicated, associated with the removal of the ill esophagus (2, 10–12).

The objective is to record the importance of continuous follow-up of these patients, the likely incidence of squamous cell carcinoma, the main known risk factors, treatment options and survival. In addition, the authors show the experience of the Service, in the follow-up of patients with previous ingestion of caustic products, in the last 40 years.

## Cancer risk

Esophageal cancer is considered the seventh most common malignant tumor, with more than 570,000 new cases reported in 2018. Among the risk factors for esophageal squamous cell carcinoma are smoking, alcohol consumption, achalasia, drinking hot drinks, deficiencies in zinc, vitamins C, E and folates, consumption of red meat, socioeconomic status and genetic factors. Esophageal adenocarcinoma is associated with reflux esophagitis, Barrett’s esophagus, obesity and alcoholism (13).

The ingestion of caustic products is a risk factor for the occurrence of esophageal cancer, and the literature reports the occurrence of the disease, approximately 30 to 40 years after the accident, mainly squamous cell carcinoma (14–18).

The first description in the literature of the association of esophageal cancer in a patient with ingestion of caustic product was made in the literature by Teleky, in 1904 (9, 11, 16, 17). Since then, patients who have ingested caustic products, at some stage of life, continue to be a matter of concern, and several cases have been reported by the authors, over the years. They need continuous monitoring, due to the exact fault of knowledge of their real incidence. In addition, there is no continuous follow-up programs in the early and chronic phases of this complication and early diagnosis of esophageal cancer.

The estimated risk in patients with a history of caustic ingestion is 1000-3000 times higher when compared to normal individuals of the same age group (1, 9, 16–18). However, the occurrence of the disease has been recorded early. Jain et al. reported the case of a 14-year-old Indian male, who accidentally ingested a caustic product and one year later developed squamous cell carcinoma in the esophagus, with cervical metastases (18).

Usually, the risk of developing cancer is 2 to 30%, between 10 and 30 years after ingestion. Alcohol abuse and smoking should be risk factors to be considered in these patients (1, 3, 5, 16, 17).

The occurrence of cancer is in the areas of narrowing and strictures in the esophagus, therefore, it is in these places that endoscopists, during the exams, need to focus their attention, in order to search for pre-neoplastic lesions (5, 9, 16)

The survival rate of patients with esophageal squamous cell carcinoma depends on the staging at the time of diagnosis and oncological and surgical treatment options (13, 19). However, the authors have reported a 5-year survival rate of 45-50% and a 10-year survival rate of approximately 15% in patients undergoing esophagectomy with neoplasms associated with caustic ingestion. The neoplasm that develops after caustic ingestion grows intramurally into the late scars of caustic esophagitis, and a small lesion worsens dysphagia in patients who commonly suffer from long-term dysphagia. In addition, diagnosis in these patients is earlier, because they have a long-term follow-up, performing esophageal dilatations and control endoscopies. And intramural fibrosis, where the neoplasm appears, does not allow rapid tumor growth, preventing the occurrence of metastases to other organs and lymph nodes (11, 16, 17, 20–23).

Ruol et al. analyzed 25 patients with esophageal scar cancer as late complication of caustic ingestion. The squamous cell carcinoma was diagnosed in 20 (80%) patients, adenocarcinoma in three (12%) and verrucous carcinoma in two patients. Esophagectomy was performed in 17 patients. The most frequent age of occurrence of carcinoma was between 40 and 70 years, with a median of 59 years (20). A possible etiology for the carcinogenesis process in the esophagus with caustic injury is the poor nutritional status of the scar tissue (21).

de Oliveira Junior et al. recorded the differentiated expression of miRNAs (miR-374 and miR-574) in esophageal mucosal biopsies of children with caustic strictures younger than 5 years, after the accident. The authors conclude that biomarker identification is a promising strategy to improve early diagnosis of esophageal cancer in caustic lesions that are at increased risk of progression (24).

Tustumi et al. performed a systematic review analyzing the risk of malignant neoplasm of the esophagus and patients undergoing esophagectomy or esophagoplasty. The authors concluded that the latency period for cancer onset ranged from 22 to 58 years and the risk of cancer in patients with caustic strictures is 701.7 - 874.1 per 1,000,000 person-years (9).

## Diagnostic tests

The digestive endoscopy must be performed, in the acute phase of ingestion of the caustic product, within the first 24 hours after the accident, in order to assess the lesion and depth extension. The Zargar classification is the most employed (7).

The endoscopic follow-up is essential in the chronic phase of the disease. There is no consensus in the literature, on the minimum time interval between endoscopic exams, however, some authors recommend starting regular follow-up about 10-20 years after the caustic accident. They suggest that endoscopic surveillance be performed every 2–3 years, but the exact intervals are unknown (16, 25–27)

Pre-cancerous dysplastic lesions are detectable through digestive endoscopy and biopsies of suspicious areas. However, routine screening is currently not recommended outside high-risk regions or for low-risk individuals. Endoscopy remains the gold standard for diagnosing dysplasia and early squamous cell carcinoma, but it is an invasive and expensive method for the health system (5, 9, 16, 17, 20, 24, 27, 28).

Pennachi et al. employed Lugol’s iodine chromoendoscopy versus Narrow Band Image enhanced endoscopy to perform biopsies in suspected areas of 38 patients with caustic stenosis for early detection of esophageal carcinomas. There were 14 confirmed lesions detected with Lugol´s solution chromendoscopy and 9 with Narrow Band Imaging. All the suspected lesions were found adjacent to stenosis. The authors concluded that the general acuity of the exams was 73% (16).

Eskander et al. analyzing the endoscopic biopsies of 100 children with caustic strictures undergoing endoscopic dilatations, of both sexes, with a mean age of 5.9 years, demonstrates evidence of chronic oesophagitis in 85%, 13% of reactive atypia in the form of severe neutrophilic inflammatory atypia and mild squamous dysplasia was diagnosed in two cases (28).

When there is already associated carcinoma, staging is necessary and the exam indicated is a chest and abdominal computed tomography (CT), to evaluate the relationship between the esophagus and the airways, and the abdominal cavity. MRI can be used, but there are no advantages. Bronchoscopy is indicated to assess invasion of the trachea and bronchi (9, 17, 19, 28–30).

Noh et al. analyzed chest CT scans of 14 patients with caustic strictures associated with malignant neoplasms that appeared on average 42 years after the caustic accident. The most common findings were eccentric wall thickening (71.4%), homogeneous esophageal wall enhancement (69.2%), periesophageal infiltration (78.5%) and enlarged mediastinal or hilar lymph nodes (14.3%) (28).

Colonoscopy should be performed when the colon is considered as an option for by-pass such as esophagocoloplasty, especially in elderly patients, who may have intestinal polyps, tumors or diverticula (2, 5, 8, 12)

## Treatment

The treatment options for patients with squamous cell carcinoma are chemotherapy (QTX), radiotherapy (RTX), chemoradiotherapy (CRT), immunotherapy, targeted therapies, endoscopic resection in early lesions and surgical treatment. Multidisciplinary or multimodal treatment is the most indicated, associating chemotherapy and radiotherapy alone, as neoadjuvants or adjuvants (13, 31). Immunotherapy has shown many therapeutic benefits in some cancer patients. The main immunotherapy options for patients with squamous cell carcinoma, anti-programmed cell death 1 ligand 1 (anti-PD-L1)/anti-programmed cell death 1 (anti-PD-1) and anticytotoxic T-lymphocyte-associated antigen- 4 (anti-CTLA-4) therapy (13).

Targeted therapy options are few, mainly employing targeting epidermal growth factor receptor (EGFR), human epidermal growth factor receptor 2 (HER2), or phosphoinositide 3-kinase/mammalian target of rapamycin (PI3K/mTOR) (13).

The surgical treatment of the association of caustic stenosis and malignant neoplasm aims at resection of the lesion, performing esophagectomy. However, the literature records a reduced percentage of esophagectomies, due to advanced disease at the time of diagnosis (**Table 1**). In most series, patients undergo radiotherapy, with or without chemotherapy associated and gastrostomies or jejunostomies, for nutritional support. The authors report 9 cases, and only one survived more than 5 years (**Table 1**).

**Table 1:** The authors who recorded the percentages of malignant neoplasms after caustic ingestion.

Publications	Number of patients with caustic stenosis	Number of cancer patients (%)	Time between caustic injury and cancer diagnosis (years)	Number of patients undergoing esophagectomy	Number of patients with survival > 5 years after esophagectomy
Hopkins et al. – USA (16) 1981	846	12 (1,4)	45,8	9	2
Mamede et al.- Brazil 2001 (5)	239	4 (1,6)	30	NR	NR
Kim et al. - Korea – 2001 (11)	54	7(13)	12	4	2
Kochhar et al. - India 2003 (15)	156	3 (1,9)	21,6	0	0
Ruol A et al. - Italy 2010 (21)	25** (3224 ***)	25 (0,8)	48	25	12
Mu et al. – China 2020 (6)	114723 *	50 (4,35)	NR	NR	NR
Chen et al.-Taiwan 2022 (32)	187	10 (5,3)	NR	NR	NR
Present study - Brazil (1979 – 2020)	415	9 (2,17)	42,5	4	1

* Ingestion of pesticides, detergents and caustics; ** Caustic ingestion; *** esophageal cancer patients. NR: not referred.

*patient recently diagnosed and yet in regimen of neoadjuvant RTX+QTX.

** patients died, up to 30 days after surgery, due to cardiac and respiratory complications.

The number of esophagectomies performed is small, it was not possible to assess either the authors’ preference for the access route for esophagectomy (transhiatal, transthoracic or videothoracoscopy), nor the most used reconstruction (5, 9, 16, 29, 30, 32, 33). Usually, the most used transit reconstruction is esophagogastroplasty, with a gastric tube. Esophagocoloplasty is an option when the stomach cannot be used. Ruol et al. reported in the analyzed series that the gastrointestinal tract was reconstructed with intrathoracic esophagogastroplasty in 8 cases, cervical esophagogastroplasty in 7 cases and cervical esophagogastroplasty in 2 cases (19).

The need or not of resection of the ill esophagus, during the reconstruction of the digestive tract, is discussed in the literature, due to the occurrence of malignant neoplasm in the dysfunctionalized organ in the mestiastinum and difficult access and subsequent diagnosis. These same authors report postoperative complications at significative rates, resulting from esophagectomy associated with bypass (11, 32).

## Conclusions

The malignant neoplasms in patients with a history of ingestion of caustic products is a real fact and has been recorded 30 to 40 years after the accident.

There is no gender preference and no more prevalent age. Tabagism and alcoholism increase the risk of cancer.

Squamous cell carcinoma is the most prevalent and occurs in areas of strictures.

The diagnosis of neoplasia is difficult and periodic endoscopic follow-up, with biopsies, is necessary for all patients with a history of caustic ingestion.

Biopsies should be obtained close to areas of strictures, where neoplasms arise.

The unacceptably high incidence of caustic ingestion in some countries and regions highlights the need to implement prevention programs and continuing adult education.

## Author contributions

All authors contributed to the article and approved the submitted version.

## Conflict of interest

The authors declare that the research was conducted in the absence of any commercial or financial relationships that could be construed as a potential conflict of interest.

## Publisher’s note

All claims expressed in this article are solely those of the authors and do not necessarily represent those of their affiliated organizations, or those of the publisher, the editors and the reviewers. Any product that may be evaluated in this article, or claim that may be made by its manufacturer, is not guaranteed or endorsed by the publisher.
